# Phenotypic and functional characterization of THP-1-derived macrophages: impact of serum and serum-free differentiation conditions on surface marker expression and immune function

**DOI:** 10.3389/ftox.2026.1843205

**Published:** 2026-07-01

**Authors:** Nour Attar, Naila Rajabli, Aline Chary, Charlotte B. A. Stoffels, Tommaso Serchi, Pamina Weber

**Affiliations:** Environmental Research and Innovation (ERIN) Department, Luxembourg Institute of Science and Technology (LIST), Belvaux, Luxembourg

**Keywords:** differentiation, functionality, macrophage, PMA, serum, serum-free, THP-1

## Abstract

**Introduction:**

Macrophages are essential components of the innate immune system, involved in inflammation and host defense. However, primary human macrophages are limited by availability, donor variability, and ethical constraints. The THP-1 human monocytic leukemia cell line is a practical alternative due to its high expansion capacity, ease of culture, and ability to acquire a macrophage-like phenotype using stimulation agent such as phorbol 12-myristate 13-acetate (PMA). To date, most laboratories generate macrophage-like cells by differentiating THP-1 cells, yet protocols vary widely between laboratories. Currently, the impact of differentiation conditions on both surface marker expression and the functional profile of macrophages remain poorly understood. Factors such as serum supplementation, PMA concentration, exposure duration, resting time, and seeding density can significantly influence macrophage behaviour.

**Methods:**

In this study, we characterized the phenotype and functionality of THP-1-derived macrophages under different differentiation conditions, with or without serum supplementation. Phenotype was assessed by flow cytometry, and functionality was evaluated through responses to the reference multi-walled carbon nanotube NM-401 following exposure using the VITROCELL® PowderX system for accurate aerosolization. Phagocytic activity was quantified using fluorescence measurements and further characterized through confocal microscopy. Inflammatory responses were assessed by measuring cytokine secretion following lipopolysaccharide (LPS) stimulation.

**Results and Discussion:**

Our findings demonstrate that differentiation conditions influence the phenotype and functional responses of THP-1-derived macrophages and highlight the importance of optimizing differentiation protocols to achieve consistent and reproducible outcomes, supporting the development of standardized, animal-free in vitro toxicity testing.

## 1 Introduction

Macrophages exhibit diverse functional phenotypes and profiles that contribute to targeted immune responses to various stimuli. Their heterogeneous and high plastic nature make them central for *in vitro* immunotoxicology and disease-relevant models (Deng et al., 2023). In the lung, alveolar macrophages represent the most abundant immune cell population, contributing to homeostasis and immune regulation at the air-liquid interface (ALI) where inhaled particles, allergens, and pathogens first encounter the host environment (Malainou et al., 2023). Their core functions, including phagocytosis, chemokine and cytokine secretion, and modulation of inflammatory responses are tightly linked to their phenotype and activation state.

One widely used *in vitro* model of the monocyte-macrophage axis in immunology and toxicology research is the human THP-1 cell line, derived from the peripheral blood of a patient with acute monocytic leukemia. In suspension culture, naïve THP-1 cells exhibit a monocyte-like phenotype and provide a practical compromise between biological relevance and experimental control, with substantially higher experimental reproducibility relative to primary human monocytes. Beyond their monocyte characteristics, THP-1 cells can be induced into macrophages through differentiation stimuli, providing a robust system to study macrophage activation and polarization in a controlled *in vitro* setting. This makes THP-1 cells a valuable alternative, especially because access to primary human macrophages is restricted by limited availability, donor-to-donor variability, and ethical considerations. Their use is highly relevant for *in vitro* models that include macrophages. For example, in our in-house developed alveolar 3D ALI cell culture model of the human airway (ALIsens®), THP-1 macrophages are added on the apical compartment of a hanging cell culture insert to mimic the airway environment, supporting the assessment of respiratory sensitization (Chary et al., 2019). The THP-1 cell line has also been adopted in regulatory-relevant assays, most prominently the human Cell Line Activation test (hCLAT), which uses THP-1 cells to quantify CD86 and CD54 as indicator of skin sensitization as described in the OECD TG442e (OECD, 2024). Building on this concept, the THP-1 activation assay has been developed to detect drug-induced immune hypersensitivity and is considered a promising first-tier screening tool in preclinical testing (Iulini et al., 2022).

A common approach to generating macrophage-like cells from THP-1 monocytes is the stimulation with phorbol 12-myristate 13-acetate (PMA) (Chanput et al., 2014). PMA acts as a protein kinase C (PKC) agonist, triggering its translocation from the cytosol to the plasma membrane, inducing diverse cellular responses like differentiation, proliferation, as well as apoptosis, and promoting the acquisition of macrophage-associated functions. PMA-differentiated THP-1 macrophages can display increased expression of macrophage markers, enhanced phagocytic activity, and robust responsiveness to secondary inflammatory stimuli (Daigneault et al., 2010). Importantly, the effects of PMA are strongly protocol-dependent, and parameters such as PMA concentration, the duration of exposure, as well as of a post-differentiation rest period can influence whether PMA-treated THP-1 cells reach a more quiescent macrophage-like state or a pre-activated phenotype, which directly affects the interpretability and comparability of measured endpoints. Despite widespread use, differentiation protocols vary widely across laboratories, resulting in inconsistent macrophage phenotype and functional responses (Mohd Yasin et al., 2022). While individual parameters such as PMA concentration and timing have been investigated in several studies, the combined effects of multiple differentiation conditions remain insufficiently characterized. In particular, the role of serum supplementation is underexplored. This distinction is important, because nutrient composition is a known determinant of differentiation and inflammatory potential. Serum supplementation introduces undefined growth factors, lipids, and binding proteins that can modulate cytokine output, phagocytic activity, and oxidative stress responses, whereas serum-free conditions may alter the basal inflammatory tone and differentiation process (Channer et al., 2025). These gaps are particularly relevant for nanoparticle toxicology, including key endpoints such as recognition and internalization impairment, reactive oxygen species (ROS) production, pro-inflammatory signalling, cytokine release and assessment of polarization dynamics (Su et al., 2025). Because mechanisms of toxicity induced by nanomaterials and chemicals differ substantially, the functional state of macrophages used in such models is highly relevant. Different exposures can drive phenotypic shifts that influence chronic toxicological outcomes, further underscoring the importance of well-characterized macrophage model.

Establishing differentiation procedures that are both reproducible and functionally meaningful is increasingly important as the demand for New Approach Methodologies (NAMs) continues to grow. These methodologies rely on standardized, human-relevant, and animal-free protocols to provide reliable *in vitro* testing. This need is emphasized by the ethical concerns associated with the use of animal-derived serum, as its production is linked to animal welfare issues, including invasive collection practices, alongside sustainability challenges and substantial batch-to-batch variability. For this reason, comparison of serum-containing and serum-free culture systems is not only biologically relevant, but also important for the further development of animal-free and regulatory-acceptable *in vitro* methods.

Based on this background, robust functional characterization of differentiated THP-1-derived macrophages is essential for interpreting immunological readouts and for improving the predictive power of NAMs. Since the evaluation of phenotypic markers alone is insufficient to conclude functional behavior, integrating surface marker panels with functional assays provides a stronger basis for comparing differentiation protocols. In the present study, we compared multiple PMA differentiation regimens that varied in PMA concentration, stimulation time, and resting period, and evaluated them under two distinct culture conditions: serum-containing and serum-free medium. We evaluated differentiation-dependent changes in cell surface marker expression, phagocytic capacity, and inflammatory responses, complemented by confocal microscopy to capture morphological and uptake-related observations. By systematically linking differentiation conditions and culture conditions to both phenotype and function, our work provides a structured optimization of THP-1 macrophages within a controlled *in vitro* system. These findings support a more reproducible and context-appropriate use of the model, improving the fitness-for-purpose in NAM-based assessments.

## 2 Materials and methods

### 2.1 Cell line

THP-1 cell line was purchased from the American Type Culture Collection (ATCC) (Supplementary Table S1) and cultured at 37 °C under 5% CO_2_ in Rosewell Park Memorial Institute (RPMI) 1640 medium supplemented with 10% fetal bovine serum (FBS). Serum-free THP-1 cells were cultured in a proprietary serum-free medium developed in house (NAMI–Non Animal Medium; patent WO 2026/032904 A^1^, (Chary and Contal, 2025), based on Opti-MEM™ Reduced-Serum Medium and ultrapure sterile water, and supplemented with defined components including Penicillin–Streptomycin, L-cysteine, L-carnosine, a chemically defined lipid mixture, nicotinamide, cytokines (Oncostatin M and Stem Cell Factor), hormones (3,3′,5-triiodo-L-thyronine, T3), divalent cations (CaCl_2_ and ZnSO_4_), and insulin–transferrin–selenium (ITS). Components were used within concentration ranges consistent with standard serum-free culture conditions. A detailed formulation and preparation protocol is currently under preparation (Chary and Contal, 2025, in preparation).

### 2.2 PMA stimulation

THP-1 cells were seeded in 6-well adherent plates at a cell density of 4 × 10^5^ cells/mL in serum-containing or serum-free culture media. Cells were stimulated directly after seeding with different concentrations of PMA: 20, 100, and 200 ng/mL. The stimulation times were 72 h or 48 h, followed by 72 h, 48 h, and 24 h resting times respectively (Table 1), after which PMA was replaced by fresh PMA-free cell culture medium (2 mL/well). PMA stock solution of 10 mg/mL was prepared by dissolving 5 mg of PMA in 500 μL ultrapure ethanol. PMA working solution of 10 μg/mL was prepared in corresponding THP-1 serum and serum-free medium.

**TABLE 1:** Summary of the experimental conditions used in this study, including varying PMA stimulation and resting times (referred to as C1, C2, C3), three PMA concentrations, and two culture media conditions (serum-containing and serum-free conditions).

Conditions	Stimulation time	Resting time	[PMA]	Culture media conditions
Condition 1 (C1)	72 hours	96 hours	20 ng/mL, 100 ng/mL, 200 ng/mL	Under serum-containing and serum-free conditions
Condition 2 (C2)	48 hours	96 hours		
Condition 3 (C3)	72 hours	24 hours		

### 2.3 Cell adherence

Supernatants and detached cells were both counted with the Countess™ automated cell counter after mixing 10 μL of cell suspension with 10 μL of 0.4% trypan blue solution (1:1 ratio) to consider the final cell yield for each condition. Cells were detached using Accutase, which was selected as a gentle detachment method compared to harsher enzymatic reagents such as TrypLE. All experiments were subjected to the same detachment procedures, ensuring any potential impact of accutase is consistent across samples and does not affect comparisons between conditions.

The percentage of adherence was calculated by subtracting the total number (N) of non-adherent cells from the starting cell number (N) seeded, divided by the total starting number of seeded cells, and multiplying by 100:

$$Adherence\;\left(\%\right)=\frac{\left(N_{seeded}{-N}_{non\;adherent}\right)}{N_{seeded}}\times 100$$

### 2.4 Cell surface marker expression using flow cytometry

Cells were detached using 2 mL of Accutase® cell detachment solution per well and incubated at 37 °C for 20 min. Detached macrophages were collected in 5 mL Eppendorf tubes, centrifuged at 300 *g* for 5 min, resuspended in 300 µL of 0.5% bovine serum MACS® BSA Solution and counted with the Countess™ before flow cytometry analysis. 1 × 10^5^ cells were transferred to a V-shaped 96-well plate pre-loaded with antibody and isotype mixes and incubated for 10 min at 4 °C in the dark. Two panels were used: panel 1 included CD62L (VB-600) and CD14 (PerCP-Vio770). Panel 2 included CD35 (APC), CD11b (VioBright-B515), and MHC-I (HLA-A-B-C) (PE-Vio615) (Supplementary Table S1). The plate was then washed with PBS and centrifuged twice, before adding the dead cell stain Sytox™ Blue (1:1000 solution in PBS) and incubating for 10 min at room temperature in the dark. Data acquisition was performed by using a BD FACSCelesta™ flow cytometer. FlowJo™ version 10 software was used for data analysis.

### 2.5 Cell exposures

Multi-Walled Carbon Nanotubes (MWCNT) NM-401 (JRCNM04001a) were kindly provided by the EU Joint Research Centre (Ispra, Italy) (Rasmussen et al., 2014). PMA-differentiated THP-1 macrophages were prepared 1 week in advance according to the *PMA stimulation* described in Materials and Methods, detached, and seeded on the day of exposure. The VITROCELL® PowderX system was used to deposit NM-401 (loading mass of 2 mg) for 15 min under high pressure, followed by a 10-min sedimentation period to allow the nanotubes to settle on culture inserts, reaching a theoretical concentration of 46.5 μg/cm^2^. After particle deposition, PMA-differentiated THP-1 macrophages (all conditions) were seeded onto the apical side of the inserts at a density of 3 × 10^4^ cells/insert in 175 μL. 600 μL of medium was added to the basolateral compartment. The inserts were inspected 4 h later, at which point an additional 100 μL of medium was added to the apical compartment to avoid drying.

### 2.6 Reactive oxygen species (ROS) assay

After 24 h exposure to NM-401, intracellular Reactive Oxygen Species (ROS) were quantified by flow cytometry. Inserts were washed with PBS and incubated with CellROX™ Deep Red Reagent for 30 min at 37 °C in the dark. Following a second PBS wash, cells were detached using Accutase® cell detachment solution for 15 min and centrifuged at 300 *g* for 5 min. Cells treated with hydrogen peroxide (H_2_O_2_) for 30 min were used as positive ROS control. The resulting cell pellets were resuspended in 200 μL Sytox blue solution diluted 1:1000 in MACS® BSA Solution to discriminate living from dead cells. Samples were mixed thoroughly, transferred to a 96-well V-bottom plate, and analyzed by flow cytometry. Unstained controls were included for each condition. Viable cells were defined as Sytox Blue-negative. Intracellular ROS levels were quantified using CellROX™ in the APC channel, and APC-positive cells were gated based on the H_2_O_2_-treated positive control. Data acquisition was performed by using a BD FACSCelesta™ flow cytometer. FlowJo™ version 10 software was used for data analysis.

### 2.7 Multiplex cytokine assay

The culture medium of the NM-401 exposed PMA-differentiated THP-1 cells was collected and centrifuged at 15,000 × g for 10 min at 4 °C to remove NM-401 fibers and cell debris. The resulting supernatant was retained for subsequent analysis. Levels of IL-8, IL-10, and TNF-α were quantified using the Bio-Plex 3D Suspension Array System from Bio-Rad according to the manufacturer’s instructions. Cytokine concentrations were determined using a standard curve generated from standards provided in the kit. Samples were measured in duplicates and data was analysed using Bio-Plex Manager software version 6.0.

### 2.8 Phagocytosis

1. pHrodo™ BioParticles™ based assay

Phagocytic activity of PMA-differentiated THP-1 macrophages was evaluated using the pHrodo™ Red *E. coli* BioParticles™ Conjugate according to the manufacturer’s protocol with minor modifications. PMA-differentiated THP-1 cells were seeded at a density of 1 × 10^5^ cells/well in 96-well plates in THP-1 growth medium and allowed to adhere for 2 h at 37 °C and 5% CO_2_. pHrodo™ Red *E. coli* BioParticles™ were suspended in HBSS (with calcium and magnesium, without phenol red), sonicated in a water bath sonicator for 10 min protected from light and added to the cells according to the manufacturer’s instructions. Control wells without BioParticles™ were included to measure background fluorescence. As a negative control, cells were pretreated for 1 h with cytochalasin B from *Drechslera dematioidea* as per manufacturer protocol and then incubated with BioParticles™. Plates were incubated for 3 h at 37 °C without CO_2_ and bottom-read fluorescence was measured at 540 nm excitation and 578 nm emission using a microplate reader (Spark 20 M, Tecan). After subtraction of background fluorescence and signal from *E. coli* particles alone, values were normalized to the fluorescence of *E. coli* particles and expressed as the percentage of phagocytosed particles.

2. Confocal microscopy on fixed cells

Phagocytic activity was further evaluated via imaging 24 h after NM-401 exposure. The culture medium was removed, and the cells were stained with Lysotracker deep red (1:2000 in THP-1 medium) for 30 min to label lysosomes. Cells were then fixed with 4% buffered formaldehyde for 15 min at room temperature. After three washes with PBS, nuclei were stained with Hoechst 33342 (1:10000 in PBS) for 15 min at room temperature on a horizontal shaker. Cells were permeabilized with 0.1% Triton-X 100 in PBS for 10 min and blocked with 1% BSA for 60 min to reduce non-specific binding. Cell membranes were stained with Rhodamine Phalloidin (1:1000 in PBS) for 45 min in the dark at room temperature. For confocal microscopy, the membranes of the hanging cell culture insert were excised from the insert using a scalpel, placed cell-side up on a glass slide, mounted with one drop of ProLong™ Glass Antifade Mountant, sealed with a cover slip, and kept at 4 °C for further analysis.

Image acquisition was performed using a Zeiss LSM 880 confocal laser scanning microscope (Zeiss, Jena, Germany) equipped with an inverted Axiovert 200 M stand. To ensure precise spatial localization within the triple-stained samples, sequential scanning was utilized to prevent spectral bleed-through between channels. Hoechst-labeled nuclei were excited with a 405 nm diode laser, while Rhodamine Phalloidin and LysoTracker Deep Red were visualized using 543 nm and 633 nm HeNe laser lines, respectively. Additionally, a reflectance mode channel was integrated into the acquisition protocol to identify NM-401 particles, after adjustment of confocal parameters with T80/R20 (transmission/reflection) ratio to optimize detection of NM-401 fibers while maximizing fluorescence transmission from cells.

Images were captured using either a ×20 objective for general field oversight or a ×63 oil immersion objective for detailed intracellular analysis. Pinhole settings were optimized for each channel to achieve a 1 Airy Unit optical section thickness. The resulting multi-channel stacks were processed and overlaid using ImageJ software (National Institutes of Health, United States) to evaluate the intracellular distribution of particles relative to the lysosomal compartment, the actin cytoskeleton, and the nucleus.

3. Confocal microscopy for live-cell imaging

For real-time visualization of particle uptake, NM-401 particles (1 mg/mL in sterile water) were added to a 24-well plate (200 µL/well) and dried completely to form a uniform layer. PMA-differentiated THP-1 macrophages were then detached and seeded on top of the NM-401 layer at a density of 1.2 × 10^5^ cells/mL (6 × 10^4^ cells in 0.5 mL of THP-1 medium). Cells were allowed to adhere for 1 h at 37 °C and 5% CO_2._ Live cell imaging was then initiated and performed overnight using a Zeiss laser scanning confocal microscope. The system was equipped with an environmental chamber maintaining 37 °C, 5% CO_2_, and high humidity. Images were acquired in transmission mode using a T-PMT detector with a ×20 objective. Laser excitation was set at 633 nm, and detector settings were optimized to minimize phototoxicity while maintaining high contrast for particle and cell visualization. The resulting time-lapse images were processed and analyzed using ImageJ software (National Institute of Health, US).

### 2.9 Statistical methods

Statistical analysis was performed using GraphPad Prism version 10.2.2, and p < 0.05 was considered statistically significant. As the flow cytometry data did not meet the normality assumptions, group comparisons were performed using the non-parametric Kruskal–Wallis test, followed by Dunn’s *post hoc* test. For experiments involving two independent variables, data was analyzed using two-way ANOVA followed by Tukey’s *post hoc* test. Multiple comparisons were visualized using a Compact Letter Display (CLD) generated in GraphPad Prism as described in the figure legends.

## 3 Results and discussion

### 3.1 Cell adherence

To first assess how the different PMA stimulation regimens and culture conditions influences the differentiation process, cell adherence was quantified as initial indicator of macrophage-like attachment and maturation.

**FIGURE 1:**
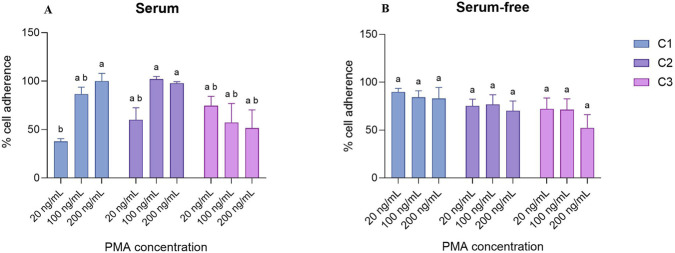
Percentage of adherent PMA-differentiated THP-1 cells, expressed as the fraction of adherent cells relative to the total number of cells seeded in serum-containing **(A)**, and serum-free conditions **(B)** The graphs show THP-1 adherence (%) across different PMA stimulation and resting conditions (C1-C3) and PMA concentrations (20–200 ng/mL). Results are shown as mean ± SEM (n = 3). Compact Letter Display (CLD) in GraphPad Prism was used to show group comparisons: groups sharing at least one letter are not significantly different, whereas groups that do not share any letters are significantly different from each other (p < 0.05).

Cell adherence was quantified to assess the extent to which THP-1 cells remained attached to the culture surface after PMA differentiation under different stimulation conditions and culture media (Figure 1). Under serum-supplemented conditions, the adherence of THP-1 cells was strongly dependent on both PMA dose and the resting time (C1-C3). The highest adherence was observed at 100 and 200 ng/mL PMA concentration, particularly under condition 2 (48-h stimulation, 96-h resting) (Figure 1A), which showed significantly more adherent cells than under condition 1 (C1) at 20 ng/mL PMA. In contrast, cell adherence under condition 3 (72-h stimulation, 24-h resting) was weaker at 100 and 200 ng/mL PMA, indicating less stable attachment and suggesting that both PMA dose and stimulation/resting duration are influencing THP-1 cell adherence. Longer recovery periods after PMA removal appear to promote more stable attachment, indicating that a short resting time may be insufficient for complete attachment under our conditions. This is consistent with previous studies showing that extended resting times (e.g., 72 h) can support differentiation outcomes such as M2-like macrophage phenotypes (Baxter et al., 2020). Interestingly, intermediate PMA concentrations may often support strong adherence without prolonged or excessive exposure. In serum-free medium, PMA-differentiated THP-1 cells showed stable cell adherence across all conditions, and no significant differences were observed (Figure 1B). This highlights the role of serum in modulating PMA-driven adherence. Serum contains adhesion-promoting proteins and growth factors (Van Der Valk, 2018) that may interact with integrins during THP-1 differentiation and enhance PMA sensitivity. By contrast, serum-free conditions bypass these serum-dependent effects and rely primarily on intrinsic, integrin-mediated adherence mechanisms.

### 3.2 Cell surface marker expression

#### 3.2.1 Effect of PMA stimulation, concentration, and serum on cell surface markers expression

To determine whether the observed differences in adherence were accompanied by changes in macrophage-like phenotype, cell surface marker expression was analyzed under the different PMA stimulation and culture conditions.

**FIGURE 2:**
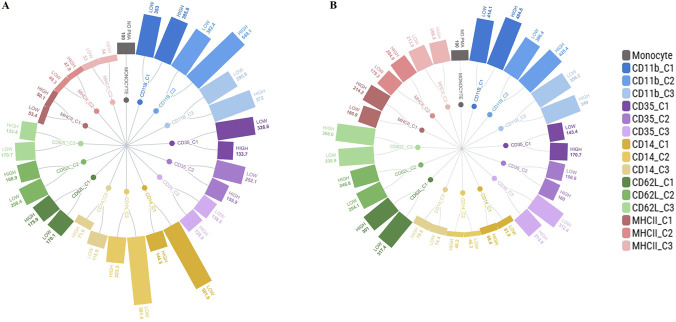
Radial bar chart representing the expression of cell surface markers in PMA-differentiated THP-1 cells across the different conditions (C1-C3), stimulated with 20 ng/mL (low PMA) and 200 ng/mL (high PMA) PMA in serum-containing **(A)** and serum-free conditions **(B)**. This presentation format allows for direct comparison of the relative expression levels of each marker across differentiation conditions. Cell surface marker expression is expressed as a percentage relative to undifferentiated THP-1 monocytes referred to as “No PMA’‘in the figure (set to 100). Results are shown as mean ± SEM (n = 3). Statistical analyses are presented in Supplementary Figure S1.

Under serum-containing conditions (Figure 2A), CD11b was consistently the most highly expressed marker across all stimulation conditions, regardless of PMA concentration or stimulation/resting duration. Its expression ranged from 295.6%–549% relative to monocytes, corresponding to a 3- to 5.5-fold increase. CD11b is known as integrin alpha M (ITGAM), a well-established and highly expressed marker of macrophage differentiation. It is involved in adhesion and immune signaling, supporting the acquisition of a macrophage-like phenotype in PMA-treated THP-1 cells (Pinto et al., 2021). CD11b, by pairing with CD18, forms complement receptor 3 (CR3), a major phagocytic receptor. CR3 binds iC3b and mediates adhesion and engulfment of complement-opsonized targets, including bacteria (Vandendriessche et al., 2021).

In the same conditions, CD35 and CD14 were predominantly upregulated at low PMA concentrations, particularly in the C1 (72-h stimulation, 96-h resting) and C2 (48-h stimulation, 96-h resting) conditions, with CD14 showing a significant induction (Supplementary Figure S1), reaching approximately a 6-fold increase relative to monocytes in low-PMA C1 and C2. CD35, also known as complement receptor 1 (CR1), is linked to the complement recognition and phagocytic functions (Török et al., 2015). Its moderate to high expressions under low PMA conditions and extended resting times reflect THP-1 maturation toward a phenotype with enhanced phagocytic and complement-binding activity. In contrast, the variable expression of CD14 observed here is consistent with previous work. Fleit and Kobasiuk (1991) reported that PMA alone did not significantly induce CD14 expression in THP-1 cells, whereas stimulation with 1,25-vitamin D3 did result in significant induction . Conversely, another study showed a marked increase in CD14 cell surface expression after 48 h stimulation with 100 ng/mL PMA (Ding et al., 2009). These discrepancies suggest that CD14 regulation in differentiated THP-1 cells is highly sensitive to culture conditions. Finally, CD62L (L-selectin), a receptor involved in leukocyte tethering and rolling, was expressed at lower levels than CD35 and CD14 but maintained a relatively stable profile across all conditions. In this study, CD62L was highly expressed in undifferentiated THP-1 cells, while PMA stimulation resulted in its downregulation, mimicking the shedding process associated with macrophage differentiation and leukocyte extravasation. Variations in CD62L levels among different PMA concentrations might therefore reflect differences in the level of maturation. MHC-II was the least expressed marker under serum conditions among all conditions tested, suggesting a low basal expression. These conditions might not induce an IFN-γ-type activation, the main driver of MHC-II, which is also associated with the cell-cycle state of the cells (Xaus et al., 2000).

Under serum-free conditions (Figure 2B), the macrophage surface marker profile differed markedly despite identical stimulation and resting times, indicating a strong influence of serum on differentiation kinetics. CD11b was significantly expressed across all conditions (Supplementary Figure S1) and reached levels comparable to those observed in serum-containing conditions, indicating that its induction is independent of serum supplementation. However, CD62L and MHC-II were expressed higher in serum-free conditions than under serum-containing conditions, showing a 3-fold and a 2-fold higher expression, respectively, and this difference was only significant for CD62L. In contrast, a different pattern was observed for CD35, which was overall less expressed in serum-free conditions. However, within this condition, the expression was highest in C3 (72-h stimulation, 24-h resting), where it reached 2- to 3-fold higher levels compared to only 1.5-fold increase in C1 and C2.

**FIGURE 3:**
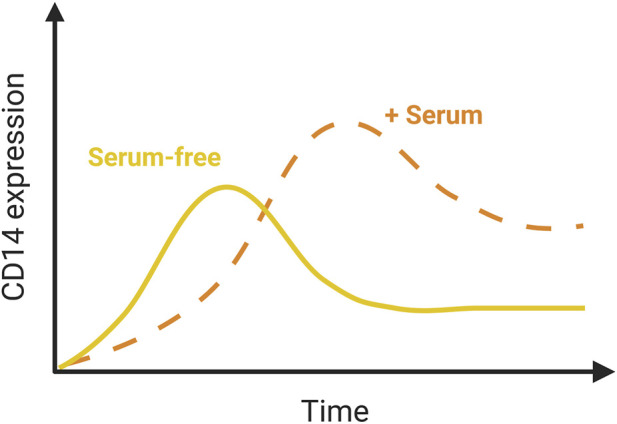
Schematic representation of the hypothesized transient CD14 transient expression in serum and serum-free conditions. Graph created with BioRender.

**FIGURE 4:**
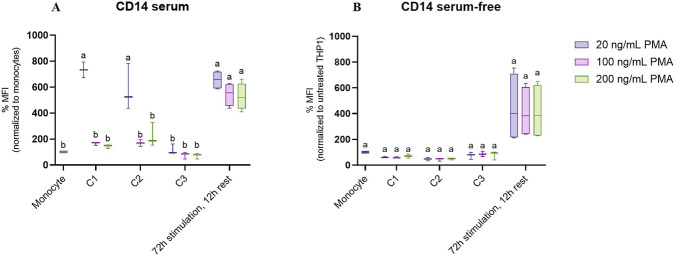
CD14 expression in PMA-differentiated THP-1 cells after 72 h of stimulation and 12 h rest, at different PMA concentrations, in serum conditions **(A)**, and serum-free conditions **(B)**. CD14 is expressed as % mean fluorescence intensity, normalized to untreated THP-1 (set at 100%). Data presented as mean ± SEM (n = 4); CLD in GraphPad Prism was used to show group comparisons: groups sharing at least one letter are not significantly different, whereas groups that do not share any letters are significantly different from each other (p < 0.05).

CD14 exhibited the lowest expression among all markers in serum-free conditions, which is in clear contrast to its strong upregulation in serum-containing low PMA conditions. One possible explanation for this differential expression is that CD14 may be transiently induced early during macrophage differentiation under serum-free conditions, potentially peaking before the earliest timepoint analyzed in this study (Figure 3). If so, its subsequent downregulation may permit the expression of a broader panel of macrophage-associated markers. In contrast, the presence of serum in the culture medium may prolong, stabilize, or delay CD14 expression, resulting in detectable CD14 at later time points but with a more restricted marker profile. Consistent with this interpretation, shortening the PMA resting time to 12 h resulted in significantly higher expression of CD14 regardless of PMA concentration in serum-containing culture conditions (Figure 4A). A similar tendency was observed in serum-free conditions, where CD14 expression was also higher compared with the later time point conditions (Figure 4B). While these findings do not establish a temporal expression profile, they suggest that the timing of analysis may strongly influence the observed CD14 levels during THP-1 differentiation.

Taken together, these findings demonstrate that serum has a substantial impact on the kinetics and pattern of THP-1 macrophage differentiation. Whereas serum-containing conditions favoured stronger late expression of CD14 and CD35, serum-free conditions promoted relatively higher expression of CD62L and MHC-II while maintaining comparable CD11b levels. Thus, although both culture conditions support macrophage-like differentiation, they appear to drive distinct maturation programs, with serum influencing not only the magnitude, but also the temporal dynamics of the marker expression. These results demonstrate that differentiation parameters, including PMA concentration, stimulation and resting time, as well as the presence and absence of serum strongly influence the resulting macrophage-like phenotype. These findings confirm the sensitivity of THP-1 cells to culture conditions and further highlight their high plasticity.

Based on flow cytometry analysis and cell adherence, PMA concentrations of 100 ng/mL and 200 ng/mL resulted in a comparable level of cell marker expression and cell adherence. The condition of 200 ng/mL did not yield any additional effect, therefore only 20 ng/mL and 100 ng/mL were selected for subsequent experiments, avoiding unnecessary exposure to excessive PMA concentrations. The C3 condition (24-h resting time) was also omitted, as both flow cytometry and adherence results indicated suboptimal differentiation and cell recovery following limited resting time post-PMA exposure.

### 3.3 Phagocytic activity of PMA-differentiated THP-1 cell

#### 3.3.1 pHrodo™ BioParticles™ based assay

**FIGURE 5:**
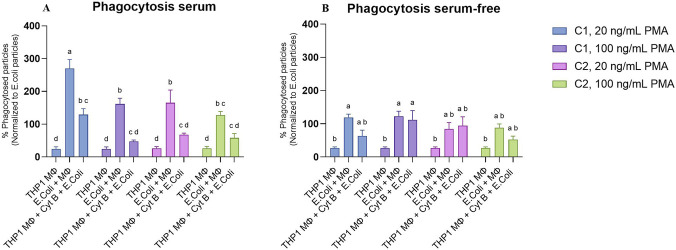
Phagocytosis quantified as mean fluorescence intensity (MFI) of internalized bioparticles, normalized to bioparticles-only control (set to 100%), after 3 h incubation with PMA-differentiated THP-1 macrophages (MΦ), in serum conditions **(A)**, and serum-free conditions **(B)**. Negative control: MΦ pretreated wit 10 μM cytochalasin B (Cyt B) for 1 h. Data shown as mean ± SEM (n = 4). CLD in GraphPad Prism was used to show group comparisons: groups sharing at least one letter are not significantly different, whereas groups that do not share any letters are significantly different from each other (p < 0.05).

To determine whether the observed phenotypic differences were accompanied by functional changes, the phagocytic capacity of PMA-differentiated THP-1 cells was assessed by measuring the uptake of fluorescent *E. coli* particles under the different stimulation conditions. Across all conditions, THP-1 macrophages displayed minimal autofluorescence, indicating that the baseline level was low and that the measured increase in fluorescence after incubation with *E. coli* bioparticles as measured in all differentiation conditions, reflected real particle internalization consistent with active phagocytosis. Under serum-containing conditions, differentiation with low PMA concentration (20 ng/mL) in C1 (72-h stimulation, 96-h resting) produced the highest phagocytic response among the tested concentrations (Figure 5A), reaching 280% normalized phagocytosis, which was significantly higher than the response observed in C1 at 100 ng/mL PMA and at both concentrations in C2 (48-h stimulation, 96-h resting), where phagocytosis remained around 150%. This result aligns with (Daigneault et al., 2010), who reported that THP-1 cells treated with PMA followed by a long resting time exhibit phagocytic capacity comparable to that of primary monocyte-derived macrophages (MDM), while continuous high-dose PMA impairs lysosome maturation and viability. Similarly, low-mid PMA differentiation upregulates the expression of genes involved in phagocytosis, including microtubule-associated protein 1 light chain 3 (LC3) and toll-like receptor genes (TLR1/6/7) thereby supporting efficient particle uptake while avoiding cellular stress associated with high-dose PMA treatment (Liu et al., 2023). To confirm that the observed fluorescence increase was due to active phagocytosis, cells were pretreated with cytochalasin B, an inhibitor of actin polymerization. Under these conditions, the percentage of phagocytosed particles decreased significantly in all groups, except the last one (C2, 100 ng/mL). These results are in line with the known mechanism of cytochalasin B, which directly inhibits phagocytosis by disrupting actin polymerization in microfilaments, as described by Malawista et al. (1971) and Axline and Reaven (1974).

Under serum-free conditions, all PMA-differentiated THP-1 cells showed a tendency toward reduced phagocytosis (Figure 5B), with a statistically significant decrease observed in C1, where cells were exposed to PMA for a longer time. This difference is likely driven primarily by the absence of FBS. Serum contains immunoglobulins (notably IgG) and complement proteins, both of which mediate phagocytosis through opsonization (Sharma et al., 2020). In particular, the complement component C3 and its cleavage products are potent opsonins that enhance particle recognition by macrophages. In addition, IgG can bind to particles, such as *E. coli* particles and facilitate their recognition via receptors expressed on THP-1 differentiated cells. In the absence of serum-derived opsonins, macrophages may instead rely more on pattern recognition receptors like the mannose receptor and other lectin-like receptors. These receptors may mediate phagocytosis via alternative endocytic mechanisms that are less dependent on actin polymerization (Kuhn et al., 2014), which could explain why cytochalasin B, an inhibitor of actin polymerization, did not effectively reduce uptake in serum-free conditions. One study reported that macrophages cultured under serum-free conditions showed overlap with an M2-associated gene expression profile, whereas macrophages cultured in the presence of FBS overlapped more strongly with M1-associated gene sets (Channer et al., 2025). This shift may further contribute to the lower phagocytic activity observed under serum-free conditions. While phagocytic activity was clearly observed, the underlying uptake mechanisms and receptor involvement were not investigated. Therefore, the data remain descriptive with respect to the pathways regulating particle internalization.

When comparing these functional data with the cell surface marker profiles, an important distinction emerges between marker expression and functional maturation. In serum conditions, CD11b was strongly expressed in both C1 (72-h stimulation, 96-h resting) and C2 (48-h stimulation, 96-h resting), indicating that both longer and shorter stimulation times induced a macrophage-like surface phenotype. However, phagocytic activity was highest in C1, showing that CD11b expression alone does not necessarily correspond to maximal functional competence. Longer stimulation and/or resting times seems to permit more complete maturation of the phagocytic machinery. This seems to include not only cell surface marker expression, but also downstream processes such as receptor activation, cytoskeletal remodelling, phagosome formation, and phagosome acidification. However, under shorter stimulation conditions, CD11b might already be expressed and present at the cell surface but not yet be functionally engaged, either due to insufficient time for receptor activation, or because the downstream machinery has not yet been completely assembled. In serum-free conditions, CD35 (complement receptor 1) expression was not the highest in C1 and C2, and phagocytic activity did not increase accordingly, despite elevated CD11b (CR3) expression. This suggests that the expression of CD35 nor CD11b alone is insufficient to determine phagocytic capacity, which more likely depends on a combination of receptor expression and activation-related signals provided by the culture environment.

Phagocytosis data show that phenotypic marker expression alone is not sufficient to define the functional state of differentiated THP-1 macrophages, indicating that acquisition of a macrophage-like surface phenotype does not necessarily mean that the cells are fully functional matured. This underlines the importance of combining marker analysis with functional readouts when establishing differentiation protocols.

#### 3.3.2 Confocal microscopy following NM-401 exposure in PMA-differentiated THP-1 cells

To further assess the functional behaviour of differentiated THP-1 macrophages under nanoparticle exposure, THP-1 cells were first differentiated according to condition C1 or condition C2 and then exposed to NM-401 particles for 24 h. Confocal microscopy and live cell imaging were subsequently used to evaluate cell morphology, particle internalization, and intracellular localization in both serum-containing and serum-free culture conditions.

**FIGURE 6:**
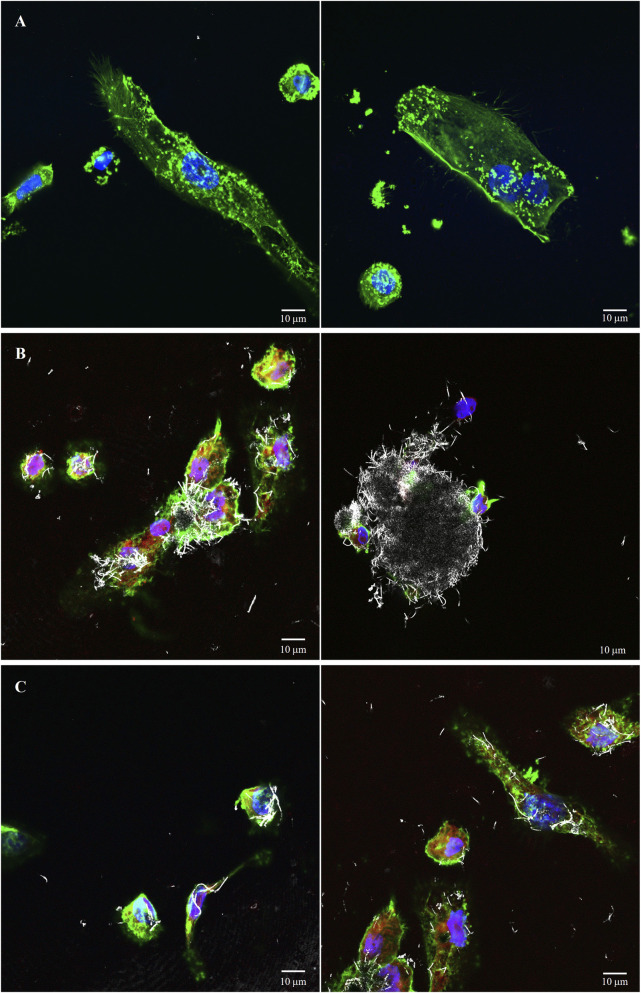
Representative confocal images of PMA-differentiated THP-1 cells in serum-supplemented medium. **(A)** Control, unexposed PMA-differentiated cells. **(B,C)** PMA-differentiated cells (condition 1 and condition 2 respectively) exposed to NM-401 for 24 h. NM-401 particles are shown in white (reflectance). Blue indicates the nuclei (stained with Hoechst 33342), and in green the cell membrane (stained with Rhodamine Phalloidin), in red the lysosomes (stained with Lysotracker). Images are acquired on Zeiss LSM 880 (×63 objective). Scale bar: 10 μm.

**FIGURE 7:**
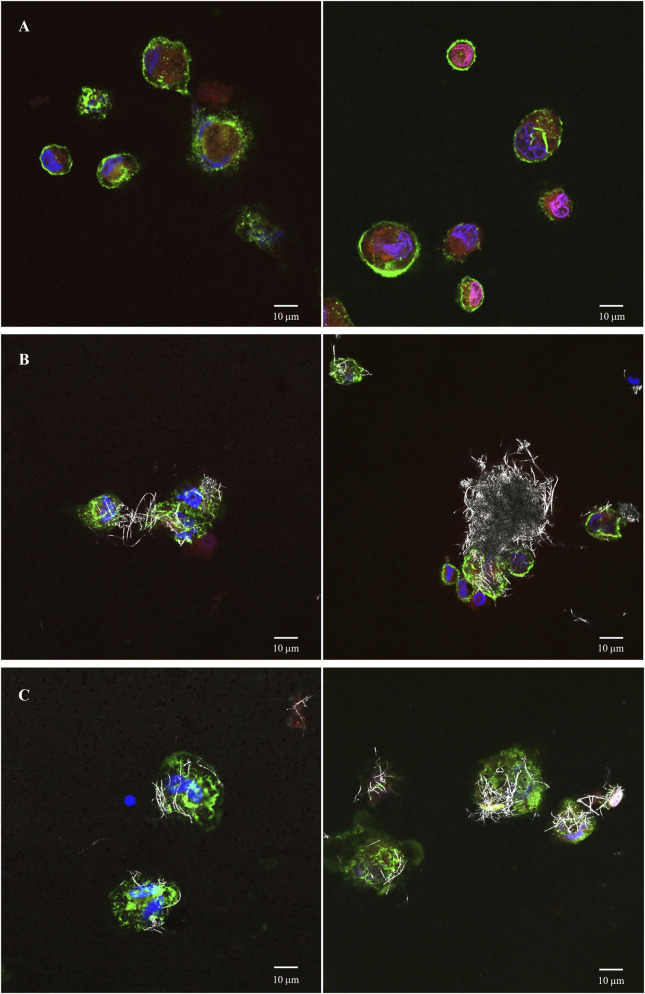
Representative confocal images of PMA-differentiated THP-1 cells in serum-free conditions. **(A)** Control, unexposed PMA-differentiated cells. **(B,C)** PMA-differentiated cells (condition 1 and condition 2 respectively) exposed to NM-401 for 24 h. Blue indicates the nuclei (stained with Hoechst 33342), and in green the cell membrane (stained with Rhodamine Phalloidin), in red the lysosomes (stained with Lysotracker), in white the NM-401 (reflectance). Images are acquired on Zeiss LSM 880 (×63 objective). Scale bar: 10 μm.

**FIGURE 8:**
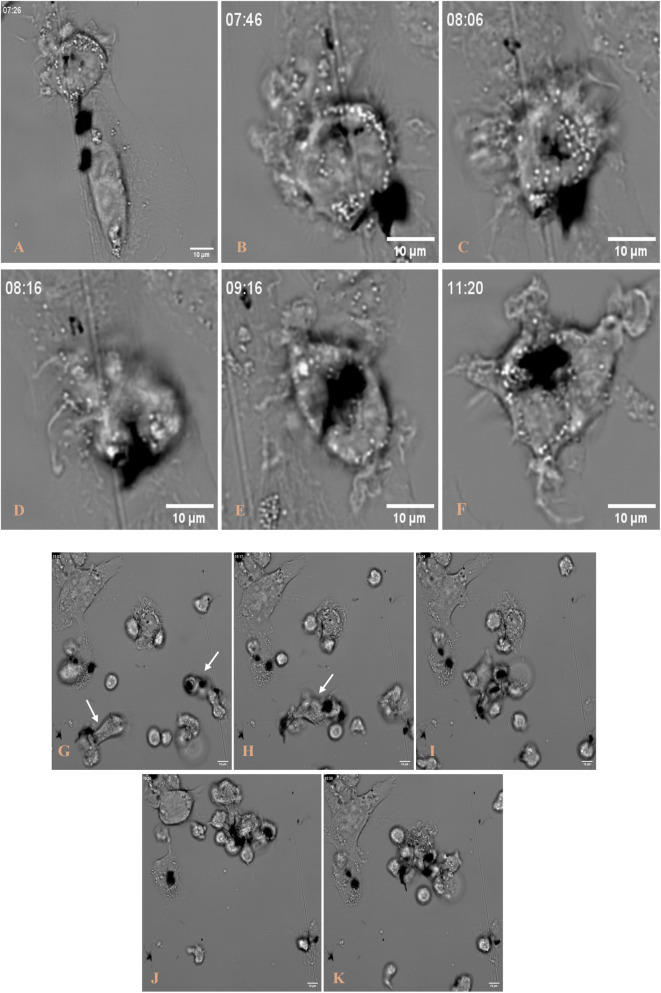
Dynamic interactions between PMA-differentiated THP-1 macrophages and NM-401 nanoparticles. **(A–F)** Progressive engulfment of NM-401 particles (shown in black) by a single PMA-differentiated THP-1 macrophage, captured every hour from 7 to 11 h post-exposure. **(G–K)** Two groups of PMA-differentiated THP-1 macrophages (white arrows) independently contact NM-401 particles **(G)**, migrate toward each other **(I)**, and integrate into a multicellular structure surrounding the particles **(J,K)**. Scale bar: 10 μm.

Unexposed PMA-differentiated THP-1 macrophages displayed distinct morphologies depending on the culture condition. In serum-supplemented medium, cells showed the typical elongated macrophage-like shape with uniform staining of the membrane (Figure 6A), which was further confirmed by scanning electron microscopy (SEM) revealing elongated cells and presence of pseudopodia protrusions at higher magnification (Supplementary Figure S2). In contrast, under serum-free conditions, PMA-differentiated THP-1 cells exhibited a more rounded-like morphology (Figure 7A), likely reflecting the absence of serum-derived adhesion-promoting proteins and growth factors. Following exposure to NM-401 nanoparticles, small fiber fragments (length <5 μm) were detected within the macrophage cytoplasm in both serum-containing and serum-free conditions, where they colocalized with lysosomal markers, indicating active intracellular processing. Both C1 and C2 differentiation protocols showed internalization of NM-401 particles within the cells (Figures 6, 7B,C), consistent with the observations from the pHrodo™ BioParticles™ phagocytosis assay. This intracellular localization is consistent with previous observations of NM-401 fibres within endosomes and the cytoplasm of murine alveolar macrophages (Erdem et al., 2023). Notably, macrophages cultured in serum-free conditions showed a reduced lysosomal signal, suggesting impaired lysosomal function or acidification (Cohignac et al., 2018), whereas lysosomes remained more clearly visible in serum-supplemented cultures despite NM-401 exposure. Live-cell imaging further revealed progressive particle uptake accompanied by active pseudopod extensions (Figures 8A–F), supporting macrophage activation. However, NM-401 fibres persisted intracellularly over several hours, consistent with the limited degradability of rigid MWCNTs. In addition to these small intracellular fragments, NM-401 also formed large micrometre-sized agglomerates (Figures 6B, 7B) that could not be fully internalized. Instead, THP-1 macrophages were observed surrounding these aggregates and interacting with protruding fibres, consistent with partial uptake or frustrated phagocytosis. This behaviour was further supported by live-cell time lapse imaging (Figures 8G–K), which showed macrophages contacting each other while attempting to engulf particle aggregates. Together, these observations indicate active biological engagement with NM-401 and confirm that differentiated THP-1 macrophages are capable of recognizing and internalizing this material, in agreement with previous reports showing scavenger receptor-mediated uptake of MWCNTs in THP-1 cells (Keshavan et al., 2021).

### 3.4 Intracellular ROS production and cytokine secretion following NM-401 exposure

Following confirmation of NM-401 internalization, intracellular ROS production was measured to assess whether particle uptake as associated with oxidative stress under the different stimulation and culture conditions. To evaluate whether NM-401 exposure induces oxidative stress in differentiated THP-1 macrophages, intracellular ROS production was measured after 24 h under the different differentiation and culture conditions. Hydrogen peroxide (H_2_O_2_) was used as a positive control, as it is a key signalling molecule relevant in ROS-dependent pathways. It is commonly used to activate Mst1 and Mst2 (mammalian STE20-like kinases 1/2), which function as critical sensors of cellular ROS *in vitro* (Wang et al., 2019).

**FIGURE 9:**
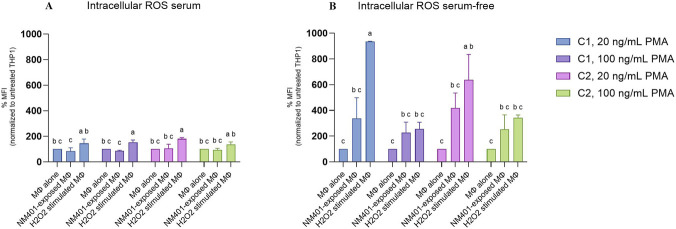
Intracellular ROS in PMA-differentiated THP-1 macrophages in **(A)** serum-containing and **(B)** serum-free conditions. ROS quantified as mean fluorescence intensity (MFI) after 24 h exposure to NM-401 and normalized to untreated THP-1 macrophages (set to 100%). Data shown as mean ± SEM (n = 3). CLD in GraphPad Prism was used to show group comparisons: groups sharing at least one letter are not significantly different, whereas groups that do not share any letters are significantly different from each other (p < 0.05).

Under serum-containing conditions (Figure 9A), exposure to NM-401 particles induced little to no increase in intracellular ROS production in PMA-differentiated THP-1 macrophages compared to untreated cells. In some conditions. The ROS levels were even slightly reduced relative to untreated control. In contrast, cells pre-treated with H_2_O_2_ showed a significant increase in the fluorescence intensity, specifically in C1 and C2 conditions at low PMA concentrations (20 ng/mL), confirming that the cells retained the capacity to exhibit a measurable oxidative response and validating the sensitivity of the assay. These finding indicate that, although NM-401 is internalized by THP-1 macrophages, it does not trigger substantial oxidative stress in the presence of serum under the present experimental conditions. These ROS data provide an insight into the functional response of the model system, as ROS production was assessed at a single timepoint, reflecting overall oxidative status but does not allow discrimination between specific intracellular sources of ROS, such as mitochondrial or NADPH oxidase-derived species.

In serum-free conditions (Figure 9B), intracellular ROS levels were generally higher than in the corresponding serum-containing cultures. However, similar to the serum-containing setting, NM-401 exposure still resulted in only a limited increase in ROS relative to untreated controls. Thus, the absence of serum appeared to increase the basal or overall oxidative state of the cells rather than to reveal a strong NM-401-specific ROS response. H_2_O_2_ again induced a clear increase in ROS signal, confirming assay responsiveness under serum-free conditions as well. This limited oxidative response may reflect the combined influence of the physicochemical properties of NM-401 and its interaction with biological components in the culture environment. It has been reported, that MWCNTs either produce or scavenge ROS depending on their physicochemical properties and the surrounding biological environment. In particular, long, rigid, needle-like MWCNTs such as NM-401 have been shown to generate unidentified free radicals (Nymark et al., 2014), which may impact oxidative stress. At the same time, biomolecules present in the exposure medium, such as albumin and other serum proteins, may reduce the surface reactivity of nanomaterials thereby reducing ROS formation. Consistent with this interpretation, the higher ROS levels observed under serum-free conditions may be explained by the absence of antioxidant protection provided by FBS. Serum contains components with ROS-scavenging properties, particularly albumin, which can bind and neutralize ROS (Grodzki et al., 2013; Pfeifer et al., 2025). In contrast, serum-free conditions provide less redox buffering capacity and potentially making cells more susceptible to oxidative imbalance. In addition, the absence of serum-derived protein corona may increase direct particle-cell interaction and enhance oxidative signalling without necessarily promoting a parallel inflammatory response (Pavlin et al., 2022).

**FIGURE 10:**
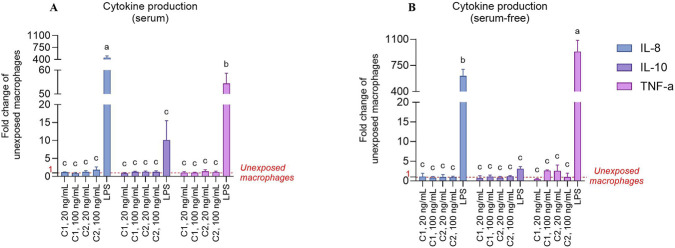
Cytokine production in PMA-differentiated THP-1 cells (C1, C2) after 24 h exposure to NM-401, expressed as fold change relative to unexposed macrophages (red line indicates the threshold value of 1) in **(A)** serum-conditions and **(B)** serum-free conditions. Data shown as mean ± SEM (n = 3). CLD in GraphPad Prism was used to indicate group comparisons: groups sharing at least one letter are not significantly different, whereas groups that do not share any letters differ significantly (p < 0.05).

Because oxidative stress and inflammatory signaling are often closely linked in macrophages, cytokine secretion was assessed in parallel to determine whether NM-401 uptake was accompanied by downstream immune activation in the THP-1 model. A similar pattern was observed for cytokine secretion (Figure 10). Across both serum-containing and serum-free conditions, NM-401 exposure induced only weak changes in IL-8, IL-10, and TNF-α release, with responses remaining below 1-fold change relative to untreated control levels. Additional cytokines (GM-CSF, IFN-γ, IL-2, IL-4, and IL-6) were measured but were below the limit of detection under these experimental conditions (data not shown), therefore subsequent analyses focused on IL-8, IL-10, and TNF-α). Although a slight increasing trend in IL-8 secretion was observed in C2 condition at 100 ng/mL PMA, this increase did not reach statistical significance. In contrast, LPS stimulation induced a strong cytokine response under both culture conditions, with fold changes exceeding 300-fold, thereby confirming that the cells retained the capacity to respond to a potent inflammatory stimulus. However, because LPS represents a strong canonical TLR4 stimulus, this positive-control response should not be taken as evidence that the THP-1 system is equally sensitive to particle-induced inflammatory signalling. Thus, the weak NM-401-induced cytokine response should be interpreted as a lack of robust cytokine induction in this specific THP-1 assay, rather than as evidence for absence of inflammatory potential.

The weak cytokine response observed after NM-401 exposure is consistent with previous findings showing that macrophage-like THP-1 cells can internalize industrial nanomaterials while releasing only low to moderate levels of IL-8 and TNF-α (Motta et al., 2024). Under serum-containing conditions, this limited inflammatory response may in part be explained by the formation of a biomolecular corona. NM-401, like other MWCNTs, can rapidly adsorb serum proteins, which may mask pro-inflammatory surface epitopes and thereby reduce immune activation, as previously reported by Zhang et al. (2019). Under serum-free conditions, despite the somewhat higher oxidative background, this was not accompanied by a corresponding increase in cytokine production. A slightly higher TNF-α signal compared with IL-8 and IL-10 was observed in C1 at 100 ng/mL PMA and in C2 at 20 ng/mL PMA, suggesting a subtle shift toward a more pro-inflammatory phenotype in the absence of serum. Nevertheless, this effect remained overall mild. Importantly, the inclusion of H_2_O_2_ and LPS demonstrates assay responsiveness to strong positive stimuli, but it does not provide a direct reference point for the sensitivity of THP-1 macrophages to NM-401-induced responses.

Taken together, these findings show that PMA-differentiated THP-1 macrophages remain responsive to appropriate oxidative and inflammatory positive controls, such as H_2_O_2_ and LPS, under both serum-containing and serum-free conditions. This confirms that the assay system was capable of detecting strong ROS and cytokine responses. However, exposure to the MWCNT NM-401 did not induce a robust increase in intracellular ROS or cytokine secretion under the tested conditions. This lack of response should be interpreted cautiously, because THP-1-derived macrophages may have limited sensitivity as cytokine producers compared with primary macrophages. Therefore, the data indicate that NM-401 did not produce a strong detectable response in this THP-1 model, but they do not exclude pro-inflammatory or oxidative bioactivity in more physiologically relevant macrophage systems or under different exposure conditions. Although confocal microscopy confirmed rapid uptake of NM-401 by THP-1 macrophages (Figure 8), particle internalization was not accompanied by marked downstream ROS generation or cytokine release in this assay. This pattern aligns with previous studies showing that certain MWCNTs elicit only modest inflammatory responses *in vitro*, despite their well-established pulmonary inflammogenicity *in vivo* (Duke et al., 2017), and that THP-1 cells are relatively poor cytokine producers compared with primary macrophages (Kermanizadeh et al., 2019). Moreover, this bioactivity of MWCNTs has been shown to depend strongly on particle length, diameter, and surface properties (Hamilton et al., 2013). As NM-401 is a rigid, high-aspect-ratio fiber, its size and shape are expected to influence its potential to perturb phagolysosomal membranes. Therefore, the weak cytokine response observed here most likely reflects a combined influence of THP-1 model sensitivity, NM-401 particle-specific properties, and culture conditions. In the absence of a primary macrophage comparator or a benchmark inflammogenic nanomaterial, these findings should be interpreted as evidence of limited detectable activation in this *in vitro* THP-1 system, rather than as definitive evidence that NM-401 lacks pro-inflammatory activity.

Discrepancies between the limited acute effects observed *in vitro* and the more pronounced responses reported *in vivo* likely reflect the greater complexity of the *in vivo* environment, where prolonged particle exposure, tissue architecture, and epithelial-immune cell interactions, extracellular matrix components, recruited immune cells, and clearance mechanisms contribute to sustained pathology (Duke et al., 2017). Together, the present findings suggest that serum mediated opsonization, protein corona formation, and redox buffering can modulate NM401–induced oxidative stress and inflammatory signaling in differentiated THP-1 macrophages. However, the dissociation between particle uptake and downstream inflammatory activation should be interpreted within the limitations of the THP-1 model and the specific culture conditions used. In particular, in the absence of a robust cytokine response after NM-401 exposure does not necessarily indicate a lack of pro-inflammatory potential, as THP-1 derived macrophages may be less sensitive cytokine producers than primary or tissue-resident macrophages. This highlights an important limitation of relying solely on oxidative stress or cytokine secretion as readouts for nanomaterial hazard assessment. Cellular responses to nanomaterials must therefore be interpreted in the context of the macrophage model, differentiation state, exposure medium, and culture conditions used. Careful control and characterization of these parameters are essential for obtaining biological meaningful and reproducible results.

In conclusion, serum-containing and serum-free conditions resulted in distinct phenotypic and functional profiles, affecting cell adherence, morphology, surface marker expression, phagocytic activity, and oxidative responsiveness. Both culture systems supported macrophage-like differentiation, but the differences observed between serum and serum-free conditions cannot be only attributed to intrinsic changes in macrophage biology. They are also likely to reflect the presence or absence of key serum-derived components, including opsonins, albumin, growth factors, lipids, and antioxidant molecules. This is particularly relevant for the interpretation of reduced phagocytic activity and altered ROS profiles under serum-free conditions. Reduced phagocytosis may at least partly result from the absence of opsonins that normally facilitate particle recognition and uptake, whereas elevated basal ROS may reflect the loss of serum-associated antioxidant buffering rather than a more physiologically relevant macrophage state. Therefore, serum-free culture should not be interpreted as more biologically faithful, but it represents a defined and potentially more reproducible culture condition that introduces specific functional trade-offs.

Accordingly, the suitability of serum-free conditions should be guided by the intended biological endpoint. Serum-free culture may be advantageous when the aim is to establish a more controlled, animal-free, and standardized differentiation protocol, because it avoids batch-to-batch variability and undefined biological effects associated with serum supplementation. In the present study, serum-free conditions supported stable adherence across stimulation conditions, increased expression of several macrophage-associated surface markers, and preserved, although partially reduced, phagocytic capacity. However, these advantages must be weighed against the associated changes in cell morphology, reduced phagocytic performance, and elevated basal oxidative state. These are not neutral effects, and they may influence the interpretation of functional endpoints, particularly particle uptake, oxidative stress, and inflammatory activation. In contrast, serum-containing conditions may remain preferable when studying serum-dependent macrophage functions, especially processes influenced by opsonization, biomolecular corona formation, particle uptake, lysosomal processing, or antioxidant buffering. Thus, the choice of culture condition should be considered an experimental variable rather than a simple optimization step.

Importantly, the present work should be viewed primarily as an optimization and characterization study within the THP-1 model system, rather than as evidence that serum-free THP-1 macrophages provide improved physiologically relevance. THP-1 cells are a transformed monocytic leukemia-derived cell line and, although they offer practical advantages such as availability, reproducibility, ease of culture, and reduced donor variability, they do not fully reproduce the phenotype or functional responsiveness of primary macrophages. Primary macrophages, such as tissue-resident macrophages (e.g., alveolar macrophages), or peripheral blood monocyte-derived macrophages (MDMs), are generally considered more physiologically representative when studying macrophage functionality, tissue-specific responsiveness, and inflammatory activity. For example, primary monocyte-derived macrophages provide a closer approximation of human macrophage biology but are affected by donor-to-donor variability, limited availability, and differences in isolation and differentiation protocols. Tissue-resident macrophages, such as alveolar macrophages, are especially relevant for inhalation toxicology and fibre-like nanomaterials such as NM-401, because they are adapted to the pulmonary microenvironment and participate directly in particle clearance and lung inflammatory responses.

However, comparative studies indicate that PMA-treated THP-1 cells are comparable to primary macrophages in several aspects. Daigneault et al. have shown that PMA-differentiated THP-1 cells display phagocytic capacity comparable to MDMs, low nitric oxide production consistent with a differentiated phenotype, and comparable expression patterns of CD14. Moreover, mitochondrial distribution and lysosomal organization resemble those of primary macrophages (Daigneault et al., 2010). In another study, MDMs from peripheral blood mononuclear cells (PBMCs) have shown a consistent and high expression of CD11 b across macrophage states, a maintained CD14 expression with variability across activation conditions, and a low MHC-II expression under baseline conditions (Lyu et al., 2023). In comparison, the PMA-differentiated THP-1 protocol used in the present study displayed a similar marker profile and aligns with observations in primary MDMs under resting conditions. However, these similarities should not obscure the important differences between THP-1-derived macrophages and primary macrophages, particularly in cytokine responsiveness, activation thresholds, donor-independent biology, and the absence of tissue-specific imprinting.

In addition to primary macrophages, induced pluripotent stem cell-derived macrophages represent an increasingly relevant human macrophage model for toxicology and mechanistic studies. Compared with primary monocyte-derived macrophages, induced pluripotent stem cell-derived macrophages can offer improved scalability, reduced donor-to-donor variability when using clonal lines, and the possibility of generating macrophages with more tissue-like phenotypes under defined differentiation conditions. Unlike THP-1 cells, they are not derived from a transformed leukemia cell line, which may make them more relevant for studies requiring a closer approximation of human macrophage biology. However, induced pluripotent stem cell-derived macrophages also require careful validation, as their differentiation state, maturation level, and tissue-specific identity depend strongly on the differentiation protocol and culture environment.

Overall, our work emphasizes the need for rigorous functional and phenotypic characterization of THP-1-derived macrophage models to improve the reliability and interpretability of *in vitro* systems used in chemical and nanomaterial hazard assessment and next-generation toxicology. The findings support the use of serum-free differentiation as a controlled and standardisable condition within the THP-1 model, but not as an unequivocal improvement in macrophage functionality or physiological relevance. Rather, serum-free culture introduces specific advantages and limitations that must be matched to the biological question being addressed. Future studies should evaluate the macrophage polarization states and compare optimized THP-1 macrophages with primary monocyte-derived macrophages, tissue-derived macrophages, and induced pluripotent stem-cell derived macrophages. Integration of well-characterized macrophage models into human lung *in vitro* systems, including air-liquid interface (ALI) co-cultures, would further strengthen their relevance for mechanistic studies.

## Data Availability

The raw data supporting the conclusions of this article will be made available by the authors, without undue reservation.
