# Supplementary material for: Phenotypic and functional characterization of THP-1-derived macrophages: impact of serum and serum-free differentiation conditions on surface marker expression and immune function
Source: Front Toxicol. 2026 Jul 1;8:1843205. doi: 10.3389/ftox.2026.1843205 (PMC13368413; doi:10.3389/ftox.2026.1843205)
Supplement: Supplementary file 3 [file DataSheet1.docx]

Supplementary Material

# Supplementary Tables and Figures

## Supplementary Tables

| **MATERIAL** | | **SUPPLIER** | **LOCATION** | **CATALOG #** |
| --- | --- | --- | --- | --- |
| **Cell Lines** | |  |  |  |
|  | THP-1 | ATCC | Manassas, VA, USA | TIB-202™ |
| **Culture Materials** | |  |  |  |
|  | RPMI 1640 Medium, GlutaMAX™ Supplement, HEPES | Gibco | Erembodegem, Belgium | 72400054 |
|  | Opti-MEM™ I Reduced Serum Medium | Gibco | Erembodegem, Belgium | 31985070 |
|  | Trypan Blue Stain | Invitrogen | Belgium | T10282 |
|  | Fetal Bovine Serum (FBS) Superior | Sigma Aldrich | Overijse, Beldium | F0615 |
|  | Phosphate Buffered Saline (PBS) | Gibco | Erembodegem, Belgium | 14190094 |
|  | Phorbol 12-myristate 13-acetate (PMA) | Sigma Aldrich | Darmstadt, Germany | 79346 |
|  | TrypLE™ | Gibco | Erembodegem, Belgium | 12604013 |
|  | Accutase - Enzyme Cell Detachment Medium | Invitrogen | Belgium | 00-4555-56 |
|  | Paraformaldehyde, 4% in PBS | Thermo Fischer Scientific | Villebon-sur-Yvette, France | J61899.AK |
|  | ProLong ™ Glass Antifade Mountant | Invitrogen | Belgium | P36982 |
|  | HBSS, calcium, magnesium, no phenol red | Gibco | Belgium | 14025092 |
| **Characterization and Functionality Materials** | |  |  |  |
|  | CD11b - VioBright-B515 | Miltenyi | Bergisch Gladbach, Germany | 130-131-718 |
|  | REA control (S), anti-human IgG1 -VioBright 515, REA293 | Miltenyi | Bergisch Gladbach, Germany | 130-113-445 |
|  | CD35 - APC | Miltenyi | Bergisch Gladbach, Germany | 130-119-511 |
|  | REA control (S), anti-human IgG1 - APC, REA293 | Miltenyi | Bergisch Gladbach, Germany | 130-113-434 |
|  | CD14 - PerCP-Vio770 | Miltenyi | Bergisch Gladbach, Germany | 130-110-523 |
|  | REA control (S), anti-human IgG1 – PerCP-Vio700, REA599 | Miltenyi | Bergisch Gladbach, Germany | 130-113-453 |
|  | CD62L VB-600 | Miltenyi | Bergisch Gladbach, Germany | 130-135-368 |
|  | REA control (S), anti-human IgG1 – Vio Bright V600, REA615 | Miltenyi | Bergisch Gladbach, Germany | 130-130-698 |
|  | MHC-I (HLA-A-B-C) PE-Vio615 | Miltenyi | Bergisch Gladbach, Germany | 130-130-041 |
|  | REA control (S), anti-human IgG1 – PE-Vio 615, REA230 | Miltenyi | Bergisch Gladbach, Germany | 130-113-451 |
|  | Sytox Blue | Thermo Fischer Scientific | Villebon-sur-Yvette, France | S11348 |
|  | MACS BSA Stock Solution | Miltenyi | Bergisch Gladbach, Germany | 130-091-376 |
|  | pHrodo™ BioParticles™ Conjugates | Thermo Fischer Scientific | Belgium | P35361 |
|  | Cytochalasin B from Drechslera dematioidea | Sigma Aldrich | Belgium | C6762-10MG |
|  | Hydrgogen peroxide solution | Sigma Aldrich | Belgium | 216763 |
|  | CellROX™ Deep Red Reagent | Thermo Fischer Scientific | Belgium | C10422 |
|  | Hoechst 33342 | Invitrogen | Belgium | H3570 |
|  | LysoTracker™ Deep Red | Invitrogen | Belgium | L12492 |
|  | Rhodamine phalloidin | Invitrogen | France | R415 |
| **Cell Culture Ware** | |  |  |  |
|  | Falcon™ 96-well, non-treated, V-shaped-bottom Microplate | Themo Fisher Scientific | Leuven, Belgium | 8772212 |
|  | Nunc™ Lab-Tek™ II chambered coverglass | Thermo Fischer Scientific | Villebon-sur-Yvette, France | 155409PK |
|  | Greiner Bio-One™ CellStar™ Polystyrene 6-well cell culture multiwell plates | Greiner | Vilvoorde, Belgium | 657165 |
|  | Greiner Bio-One™ CellStar™ Polystyrene 12-well cell culture plates | Greiner | Vilvoorde, Belgium | 665165 |
|  | 12-well Insert 1.0 µm PET clear | CellQART | Northeim, Germany | 9311012 |
|  | Nunc™EasYFlask™ Cell Culture Flasks 75 cm*2* | Thermo Fischer Scientific | Villebon-sur-Yvette, France | 156499 |
|  | Greiner Bio-One μClear™ Bottom 96-well | Greiner | Vilvoorde, Belgium | 655096 |
| **Equipment** | |  |  |  |
|  | Countess™ automated cell counter | Thermo Fischer Scientific | Belgium | A49865 |
|  | BD FACSCelesta™ Cell Analyzer (flow cytometer) | BD Biosciences | Erembodegen, Belgium |  |
|  | Bio-Plex® 3D suspension array system | Luminex Corporation | Oosterhout, The Netherlands |  |
|  | PowderX | VITROCELL^®^ | Waldkirch, Germany |  |
|  | Spark 20 M (Fluorescence microplate reader) | Tecan | Mechelen, Belgium |  |
|  | Zeiss LSM 880 with inverted Axiovert 200 M microscope (version 14.0.29.201). | Zeiss | Germany |  |
| **Software** | |  |  |  |
|  | FlowJo™ version 10 software | FlowJo LLC | Ashland, OR, USA |  |
|  | GraphPad Prism version 10.2.2 | GraphPad Software Inc. | San Diego, CA, USA |  |
|  | BD FACS DIVA software | BD Biosciences | Erembodegen, Belgium |  |
|  | Zen 2.3 SP1 FP3 (black) | Zeiss | Germany |  |
|  | Bio-Plex Manager™ software version 6.0 | Luminex Corporation | Oosterhout, The Netherlands |  |

Supplementary Table 1: List of cell lines, cell culture media, reagents, materials, antibodies, equipment and software used in this study, including supplier and catalog number.

## Supplementary Figures


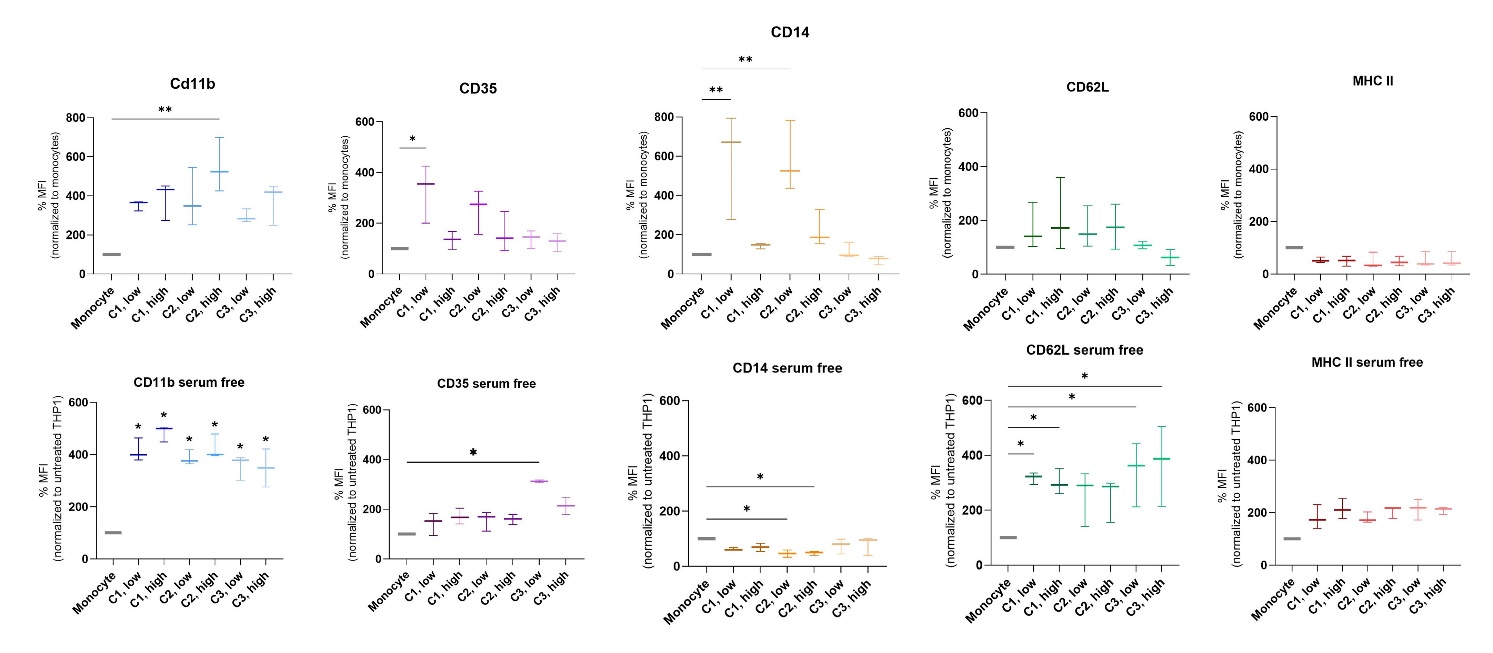


Supplementary Figure 1: Kruskal-Wallis test results comparing expression levels of cell surface markers in PMA-differentiated THP-1 cells across the different conditions (C1-C3), stimulated with 20 ng/mL (low PMA) and 200 ng/mL (high PMA) PMA in serum and serum-free conditions. Data represents median fluorescence intensity (MFI) normalized to untreated THP-1 cells from n = 3 independent experiments, *p < 0.05, **p < 0.01 (Dunn's post-hoc).


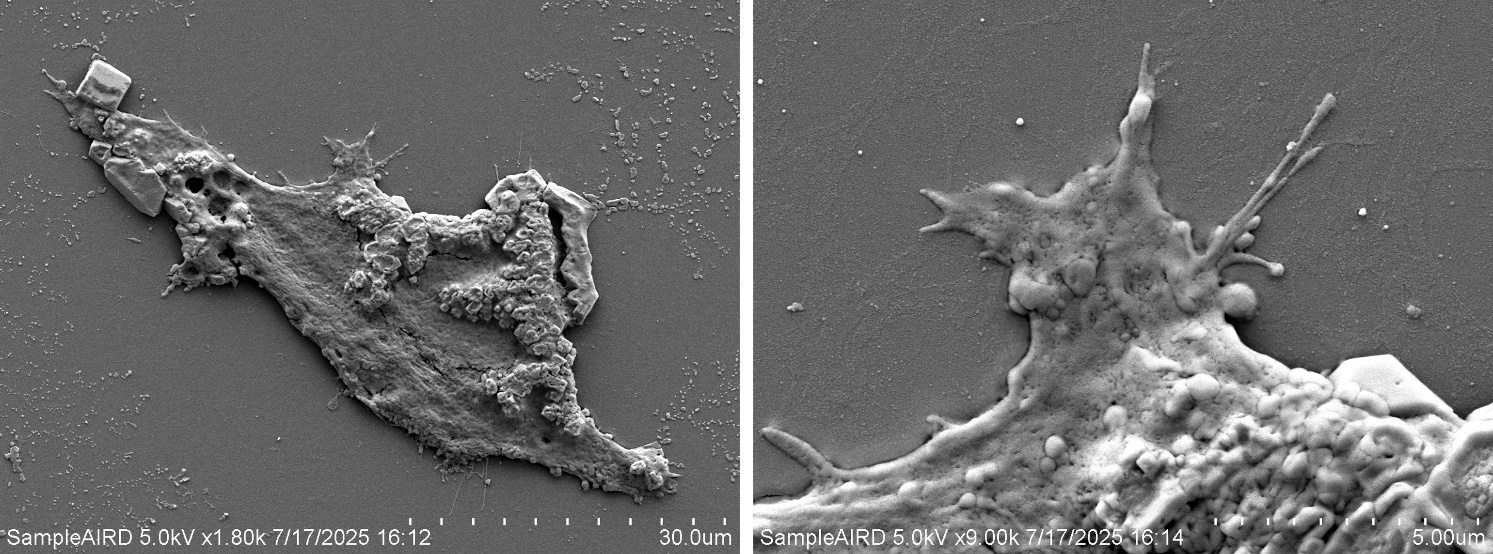


Supplementary Figure 2: Scanning electron microscopy (SEM) of macrophages cultured on coverslips. Macrophages (1 × 10⁵ cells) were seeded onto 12 mm glass coverslips placed in 6-well plates (2 mL medium/well) and allowed to adhere overnight. Cells were then washed with PBS and fixed with 4% formaldehyde at room temperature for 15 minutes. Following fixation, samples were washed with distilled water and air-dried completely to minimize salt crystal formation. Dried coverslips were mounted onto SEM pin stubs using conductive adhesive and sputter-coated with silver to enhance surface conductivity and prevent charging during imaging. SEM imaging was performed at an accelerating voltage of 5.0 kV. Representative images were acquired at ×1,800 magnification (30 µm scale bar) and ×9,000 magnification (5 µm scale bar).


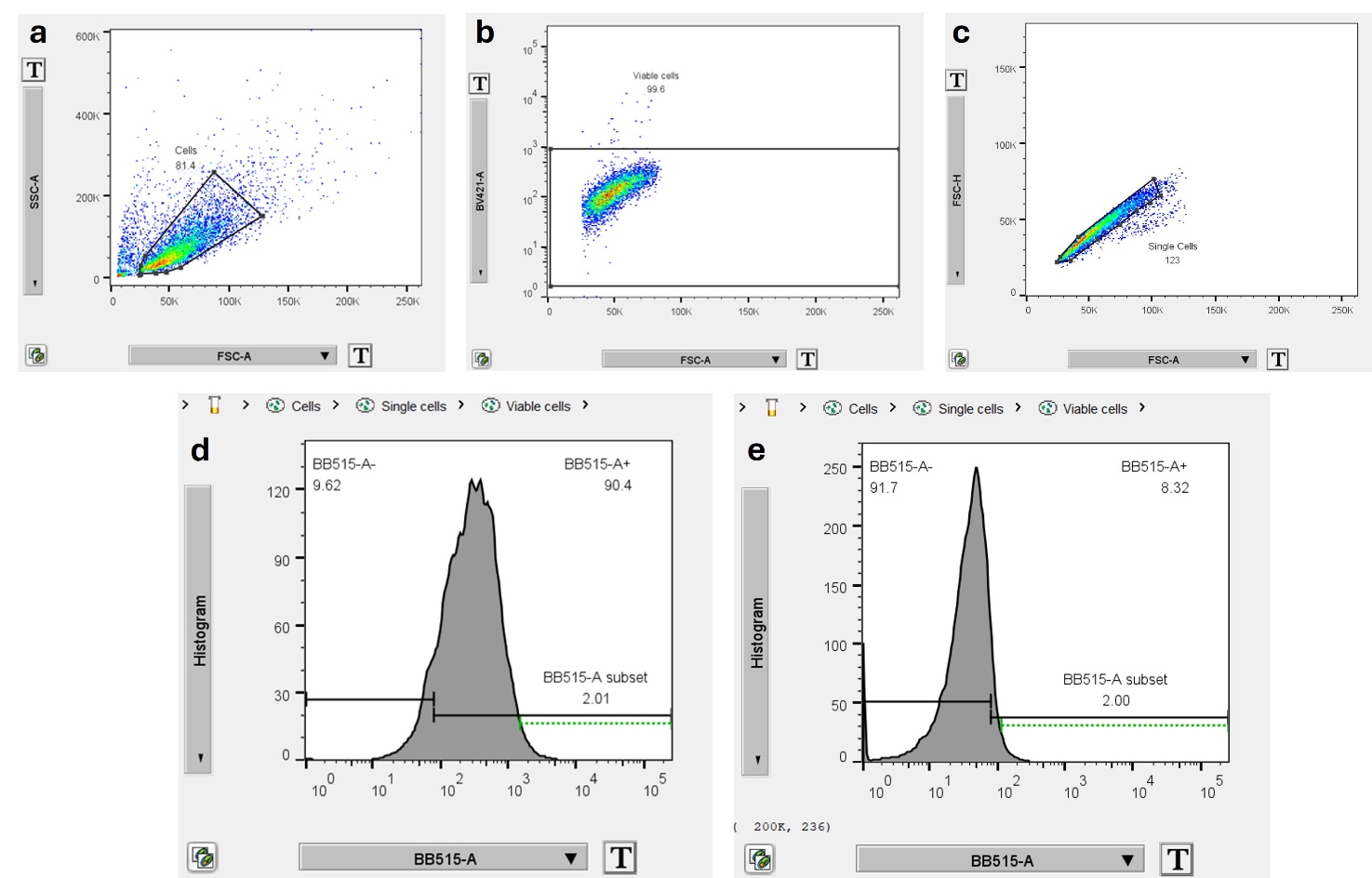


Supplementary Figure 3: Flow cytometry gating strategy for THP-1 cells.

a: Initial gating of the main cell population based on forward and side scatter to exclude debris.
b: Gating of viable cells using BV421-A (SYTOX™ Blue), where viable cells are defined within the fluorescence intensity range corresponding to y-values between 10⁰ and 10³.
c: Identification of singlets by gating on FSC-A versus FSC-W (or FSC-H) to exclude doublets and aggregates.
d, e: Gate setup for cell surface marker analysis in a representative example shown for CD11b (BB515). Gates were defined using isotype controls by setting the threshold at the 98^th^ percentile of fluorescence intensity for the fluorochrome of interest. This gate was then applied to antibody-stained samples in d: PMA-differentiated THP-1 cells under low PMA conditions (20 ng/mL) and e: undifferentiated THP-1 monocytes.


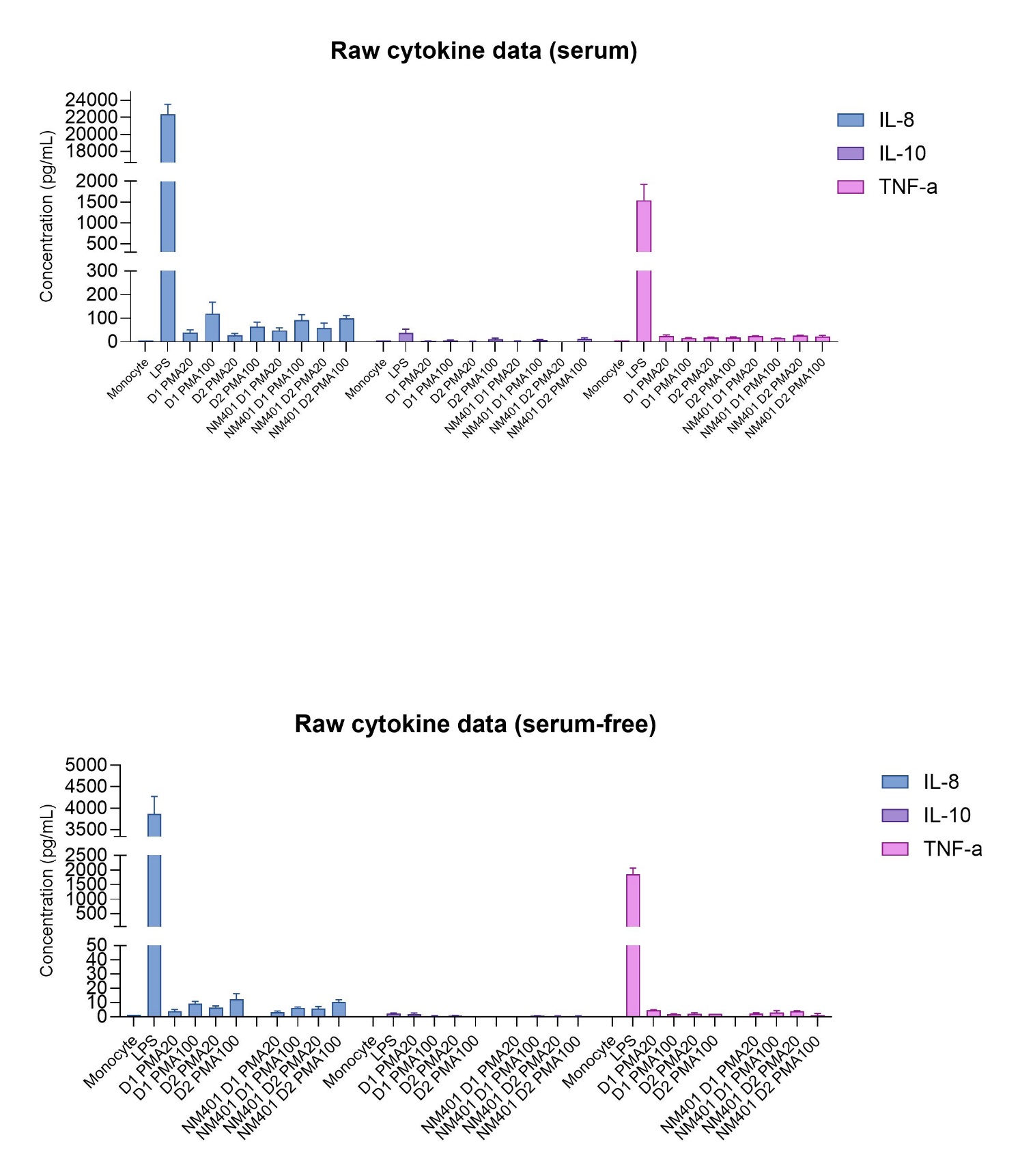


Supplementary Figure 4: Observed concentrations (pg/mL) of IL-8, IL-10, and TNF-α measured in cell culture supernatants under serum-containing and serum-free conditions. Data are presented as absolute cytokine levels to complement fold change analyses. Cytokine quantification was performed using , and values represent mean ± SEM of 3 independent experiments.
